# Monitoring the T-Cell Receptor Repertoire at Single-Clone Resolution

**DOI:** 10.1371/journal.pone.0000055

**Published:** 2006-12-20

**Authors:** Hendrik P.J. Bonarius, Frank Baas, Ester B.M. Remmerswaal, René A.W. van Lier, Ineke J.M. ten Berge, Paul P. Tak, Niek de Vries

**Affiliations:** 1 Division of Clinical Immunology and Rheumatology, Academic Medical Centre, University of Amsterdam Amsterdam, Netherlands; 2 Department of Neurogenetics, Academic Medical Centre, University of Amsterdam Amsterdam, Netherlands; 3 Department of Experimental Immunology, Academic Medical Centre, University of Amsterdam Amsterdam, Netherlands; 4 Division of Nephrology, Department of Internal Medicine, Academic Medical Centre, University of Amsterdam Amsterdam, Netherlands; Université de Toulouse, France

## Abstract

The adaptive immune system recognizes billions of unique antigens using highly variable T-cell receptors. The αβ T-cell receptor repertoire includes an estimated 10^6^ different rearranged β chains per individual. This paper describes a novel micro-array based method that monitors the β chain repertoire with a resolution of a single T-cell clone. These T-arrays are quantitative and detect T-cell clones at a frequency of less than one T cell in a million, which is 2 logs more sensitive than spectratyping (immunoscope), the current standard in repertoire analysis. Using T-arrays we detected CMV-specific CD4+ and CD8+ T-cell clones that expanded early after viral antigen stimulation *in vitro* and *in vivo*. This approach will be useful in monitoring individual T-cell clones in diverse experimental settings, and in identification of T-cell clones associated with infectious disease, autoimmune disease and cancer.

## Introduction

T cells are key players in the antigen specific immune responses. Antigen specificity is provided by the T-cell receptor (TCR), which is unique for each T-cell clone. Upon antigen recognition, individual T cell clones generally expand and acquire differential effector properties. Although the number of potential TCRs has been estimated at 10^15^ different α/β combinations [1], the actual αβ TCR repertoire per individual is estimated to include 10^6^ different β chains [2], each pairing with a limited number of α chains [3]. There is no rapid technology available that can sensitively and quantitatively monitor this highly diverse T-cell receptor repertoire.

Current technology for screening the TCR repertoire for expanded T-cell clones relies on ‘spectratyping’ [4], often referred to as immunoscope, and/or individual cloning and sequencing of a sample of the T-cell population [2], [5]–[9]]. In spectratyping analysis, PCR amplified TCR DNA is separated on size of the CDR3 region. This approach separates the TCRβ repertoire in approximately 230 fractions, resulting from the use of ∼23 primers for all functional Vβ families and about 8 different CDR3 lengths per Vβ [10]. A higher resolution can be attained when V- and J-region primers are used; however, this requires 23 · 13 individual PCR reactions, and results in a resolution of approximately 23 · 13 · 8 peaks (**Table S2**).

Spectratyping itself generally does not identify individual T cell clones, and is therefore often followed by repetitive cloning and sequencing. Clonal peaks identified in the spectratype patterns are sequenced, typically 10^2^ clones and maximally 10^4^ clones per sample [2], [5]–[9]] in previous publications. The sensitivity of this combined approach depends on the sensitivity of spectratyping for identification of clonal peaks, and on the number of T-cell-receptor rearrangements cloned and sequenced. Thus, although the combination of spectratyping with sequencing can attain sufficient resolution to analyze TCR diversity, the approach is laborious and time consuming as it requires PCR amplification, isolation of individual bands based on DNA size, purification, followed by repeated cloning and sequencing.

Here, we explore a novel approach which exploits the high capacity of DNA microarrays to monitor the expression of many T-cell receptor rearrangements in parallel. At present, it can be used to follow T cell responses in cases where type of Vβ/Jβ and length of Jβ-gene segment are available, e.g. from prior immunoscope (spectratyping) experiments. The feasibility of this approach is shown, and validated both in vitro and in vivo. We show that T arrays quantitatively monitor the expansion of T-cell clones after viral infection with high sensitivity (1 in 10^6^ cells), and with sufficient resolution to identify single clones in a background of polyclonal peripheral blood T-cells. While at present it allows monitoring a Vβ/Jβ-specific fraction of 0.03% of the T-cell receptor repertoire on a single 4000-spot slide, the microarray-based method can be scaled up to monitor and screen a large pool of the T-cell repertoire for dominant clonotypes. We envision that this sensitive and rapid technology will be useful for monitoring and screening of clonal T-cell expansions for many applications in medical research.

## Results

### Creating single-clone resolution

**Figure 1:**
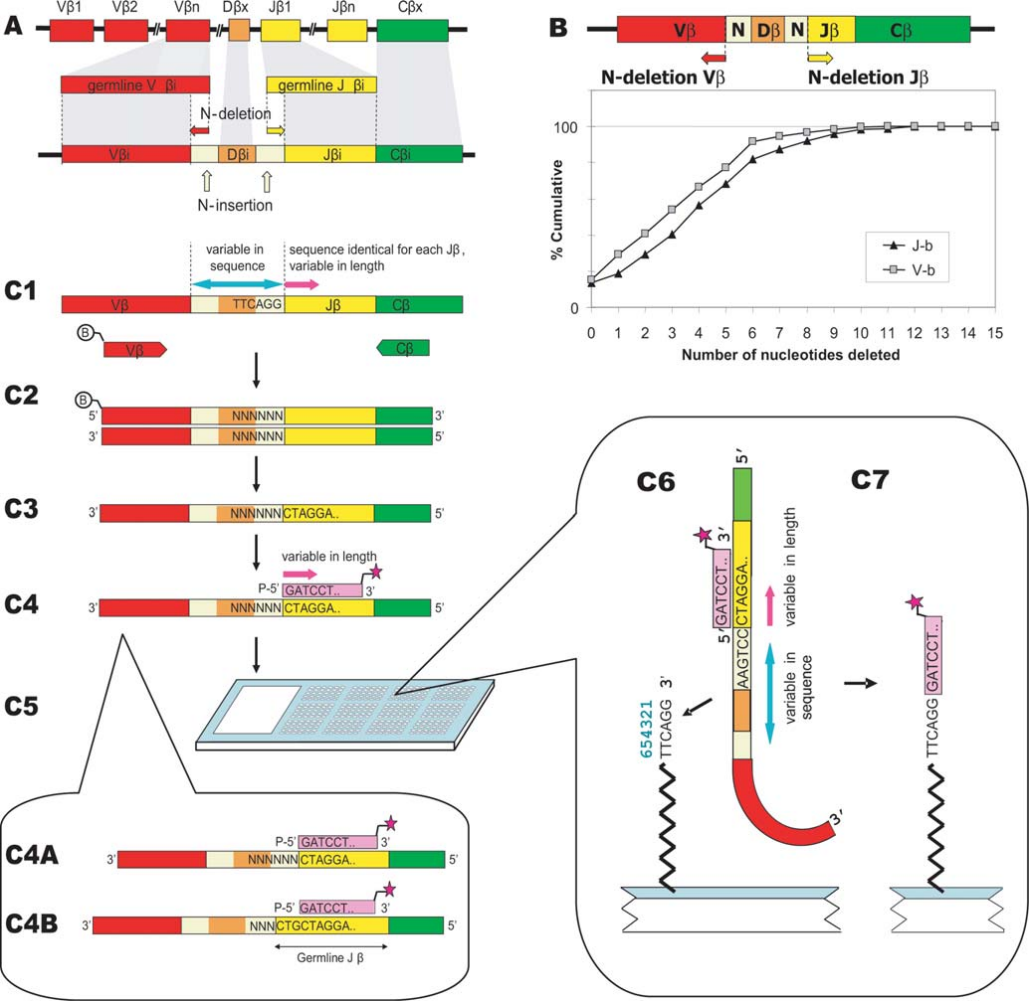
The T-array protocol. **(A)** During development, VDJ recombination causes enormous variability in TCRβ chain by randomly selecting different combinations of 23 V, 2 D, and 13 J gene segments, by nucleotide insertion (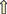), and by nucleotide deletion from V (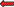), D, and J (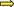) genes. This results in a diversity of an estimated 10^6^ different β chains per individual. **(B)** N-deletion causes shortening of the Vβ and Jβ segments. The number of nucleotides deleted from Vβ and Jβ germline DNA is limited. N-deletion of 192 published TCRβ mRNAs was determined. The figure shows the cumulative percentage of CDR3βs for the number of nucleotides deleted. TCRβ's with n nucleotides deleted represent approximately 10% of the repertoire if n = 0 to 6, and 5%, if n = 7 to 9. **(C)** The T-array protocol: **(C1)** cDNA from T-cells is generated. **(C2)** CDR3β regions are PCR amplified using biotinylated Vβ-specific (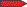) or Vβ-generic primers (not shown here). **(C3)** Biotinylated strands are removed after alkaline denaturation using streptavidin-coated beads. **(c4)** Single-strands of polyclonal TCRs are aliquoted and hybridized to fluorescently labeled annealers (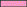) complementary to the NDN-adjacent end of a Jβ gene. A specific number of Jβ-gene nucleotides (n) is deleted for each annealer, accounting for N-deletion during the VDJ recombination process. Insert (C4): Each annealer will hybridize to TCRβ rearrangements where n nucleotides are deleted from the Jβ-germline gene segment (**C4A**) or where less than n nucleotides are deleted (**C4B**). **(C5)** The annealer-hybridized fractions are loaded on universal hexamer arrays for **(C6)** T-cell-clone-specific ligation and, **(C7)** subsequently washed, scanned and analyzed.

To create adequate resolution between different potential TCRs we focussed on the highly variable complementarity determining region 3 (CDR3) of the TCR beta chain. This region consists of one out of 40–48 functional Vβ and one out of 13 functional Jβ segments, joined by Dβ gene segments [11]–[13]. The CDR3 is generated during VDJ-recombination by random deletion and addition of nucleotides at the V-, D-, and J-junctions [1] and produces the hypervariable NDN region, which can be used as a signature for each TCR (**Fig. 1A**). We developed a T-array protocol (**Fig. 1C**) to interrogate the first six nucleotides of the NDN region and the length of the Jβ-gene segment. Resolution is created in three subsequent steps by: *i*) Vβ-specific PCR amplification of the CDR3β (**Fig. 1C1 and C2**), *ii*) hybridization of a labelled oligonucleotide (“annealer”) specific for the Jβ-family and for the number of Jβ-nucleotides deleted (**Fig. 1C4**), and (*iii*) a ligation reaction specific for the first six nucleotides of the hypervariable NDN region on a universal hexamer microarray [14], encoding all permutations of a hexamer nucleotide (**Fig. 1C5**). In this way, the hexamer sequences on the array complementary to the first six nucleotides of the NDN region of a T-cell clone are ligated to the fluorescent annealer probes (**Fig. 1c6–7**). The fluorescent signal of each hexamer sequence on a single microarray chip, quantitatively reflects the expansion of a certain T-cell clone. It should be noted that an annealer designed for a Jβ gene with n nucleotides deleted from the germline sequence will also give a signal for TCRs with less than n nucleotides deleted. The latter TCRs will reveal part of the germline sequence of Jβ in their hexamer sequences (**1C4B**).

The resolution of this T-array protocol depends on the number of Vβ and Jβ segments, the size of the microarrays, and the number of Jβ-nucleotides deleted. To predict the potential resolution of the assay we analyzed the distribution of N-deletion in a random selection of 192 published CDR3β mRNA sequences (**Table S1**). For 99% of the sequences a maximum of 10 nucleotides is deleted from Vβ genes, and a maximum of 11 nucleotides from Jβ genes (**Fig. 1B**). Within these limits, an almost uniform distribution of the TCRs was observed over the number of nucleotides deleted. This enabled us to predict the potential resolution of the assay. Although the theoretical size of the TCR repertoire is estimated at 10^15^, extensive cloning experiments have shown that within one individual the beta-chain repertoire contains approximately 10^6^ unique sequences [2], each of which pairs with a limited number of α chains [2], [3]. Based on these numbers we estimate that after Vβ/Jβ-specific amplification on average 10^6^/1.4 · 10^7^ = 0.07 CDR3β sequences from the complete repertoire of a human individual will ligate to a single sequence on the universal hexamer microarray (see also **Table S2C**). In theory, the assay should therefore have sufficient resolution to detect single CDR3β sequences.

### Testing sequence specificity and validity

**Figure 2:**
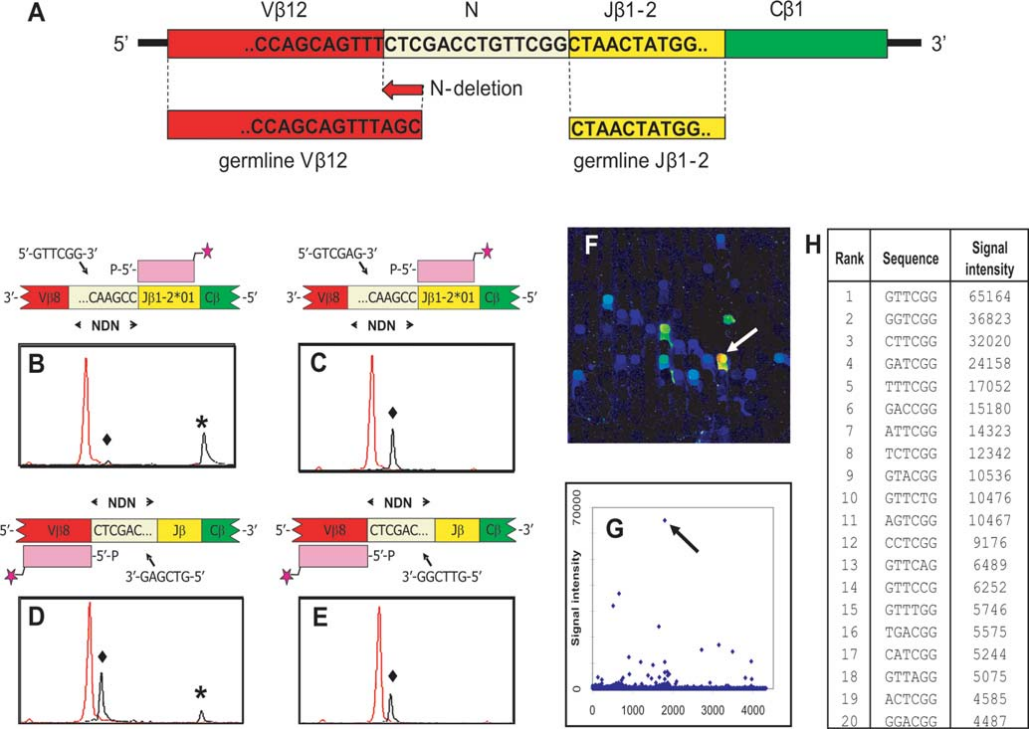
Ligation experiments with Jurkat CDR3β amplicons. **(A)** VDJ rearrangement of Jurkat TCRβ. In this CDR3 sequence, there are 3 and 0 nucleotides deleted form the germline Vβ and Jβ, respectively. **(B–E)** Capillary-electrophoresis chromatograms showing length and signal of fluorescent annealers (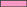). **(B)** A hexamer (GTTCGG) complementary to the Jurkat 3-end NDN sequence elongated (*) the Jβ1-2-specific annealer (♦). **(C)** The non-complementary 5′-end hexamer failed to cause elongation. Black peaks: Cy5 signal; Red peaks: FAM signal of size standards. **(D, E)** Similarly, a Cy5-labeled oligonucleotide complementary to the 3′ coding end of Vβ12, was elongated (*) only in the presence of the hexamer GTCGAG which is complementary to the 5′-end of the NDN sequence. **(F)** Detail of universal hexamer array after ligation of a Cy5-labeled oligonucleotide complementary to the 5′ coding end of Jβ1-2 in the presence of the antisense strand template of Jurkat CDR3β. **(G)** Signal intensities of all 4096 spots of the same array experiment. The arrow in (F) and (G) indicates the microarray spot with sequence GTTCGG. **(H)** List of 20 strongest spots with their hexamer sequence and Cy5-fluorescent signal.

The specificity of the protocol was tested using the T-cell clone Jurkat E6-1, for which the CDR3 is known. After PCR amplification of the Jurkat CDR3β region, we isolated the antisense strand and hybridized it to a fluorescently labelled oligonucleotide encoding the NDN-oriented end of the Jβ1-2 sequence. Specificity of the ligation reaction for Jurkat NDN sequence was then tested with hexamers either complementary or not complementary to this NDN sequence (**Fig. 2B–C**). Only in the presence of the complementary hexamer sequence (5′-GTTCGG-3′) the annealer oligonucleotide was elongated, indicating that the ligation is sequence specific for the Jurkat CDR3β. Similarly, when the sense strand was used as a template, the annealer was elongated only with the hexamer sequence complementary to the 5′-end of NDNβ (**Fig. 2D–E**). When tested on a universal hexamer array, out of all 4096 possible sequences the Jurkat NDN sequence GTTCGG gave the strongest signal (**Fig. 2F–H**). This shows that the protocol is sequence specific for the T-cell clone analyzed. However, some other spots did produce positive signals, albeit at much lower signal intensities, notably if the encoded sequence was identical in the 3′-end nucleotides (NNTCGG). This suggests that, apart from the strongest signal at hexamer GTTCCG, the Jurkat NDN sequence ligated to hexamers with a 5′-end mismatch.

### Determination of sensitivity T-array

**Figure 3:**
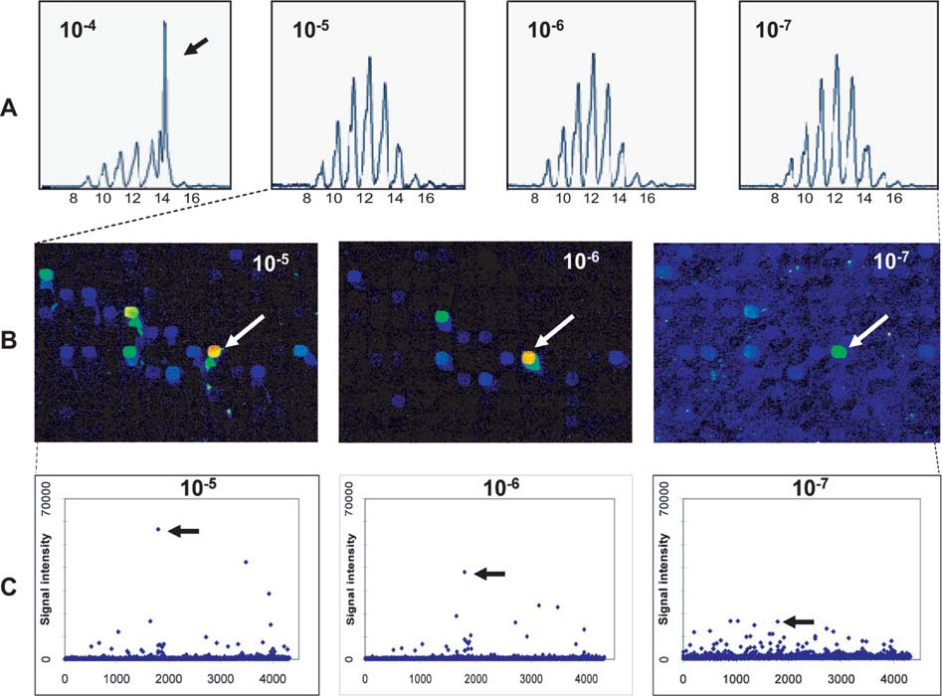
Spectratyping and T-array for Jurkat T-cell clone mixed with peripheral blood CD4^+^ T-cells. Jurkat cells were added in different dilutions to a background of peripheral blood CD4^+^ T-cells. **(panel A)** CDR3 spectratyping with Vβ12-specific primer. The arrow indicates a length identical to 14 amino acids, which is the length of the Jurkat CDR3β. **(panel B)** A detail of the T-array scans. White arrows indicate hexamer sequence GTTCGG, which is complementary to the first six nucleotides Jurkat NDNβ region. **(panel C)** Signal intensities of all 4096 spots of the T-array. Black arrows indicate hexamer sequence GTTCGG.

Having shown that the T-array is specific for the NDN sequence of the analyzed T-cell clone we then compared the sensitivity of the assay to that of spectratyping by diluting decreasing proportions of Jurkat cells in a background of peripheral blood CD4^+^ T-cells. Semi-quantitative PCR showed that TCR transcripts in Jurkat cells were not more abundant than in CD4+ cells obtained from a healthy blood donor (**Fig. S1**). For immunoscope, Jurkat/CD4+ mixtures were then PCR amplified with a Vβ12-sense primer and a fluorescamine-labelled Cβ reverse primer and size separated by capillary electrophoresis (**Fig. 3A**). As expected, the size difference between DNA peaks was 3 bp and the peak signals were normally (Gaussian) distributed [5]. Only in the case of a dilution of 1 Jurkat cell per 10^4^ CD4^+^ blood cells, the peak associated with Jurkat CDR3β length (14 amino acids) is 47% of the total peak area. At dilutions of 1 in 10^5^ to 1 in 10^7^ the peak associated with 14 amino acids is 7–8% of the total peak area, and the peak distribution remains normally distributed, indicating no dominance of any CDR3 length in the Vβ12 compartment. These results show that the sensitivity of spectratyping is approximately 1 T-cells in 10^4^ cells, which is in agreement with other reports [15].

For T-arrays, the antisense strands were isolated and hybridized to the Jβ-1-2-specific, Cy5-labeled oligonucleotide mentioned earlier and ligated on a universal hexamer microarray (**Fig. 3B–C**). In the case of a 1 in 10^5^ to 1 in 10^7^ Jurkat/CD4^+^ ratio, the hexamer sequence GTTCGG, which is complementary to the 3′ end of Jurkat NDN region, was quantitatively picked up (**Fig. 3C**). In the case of a 1 in 10^7^ dilution, the GTTCGG signal was similarly intense as the hexamer spots TGTCGG and CTTCGG. These sequences only differ in the nucleotides at the terminal end of the ligation product, suggesting that these are 5′ mismatch ligations of the Jurkat sequence. These results show that in this format of the T-array protocol, individual T-cell clones are picked up with a sensitivity of 1 clone in 10^6^ T-cells.

### Detection of expanding T-cell clones after viral antigen stimulation

**Figure 4:**
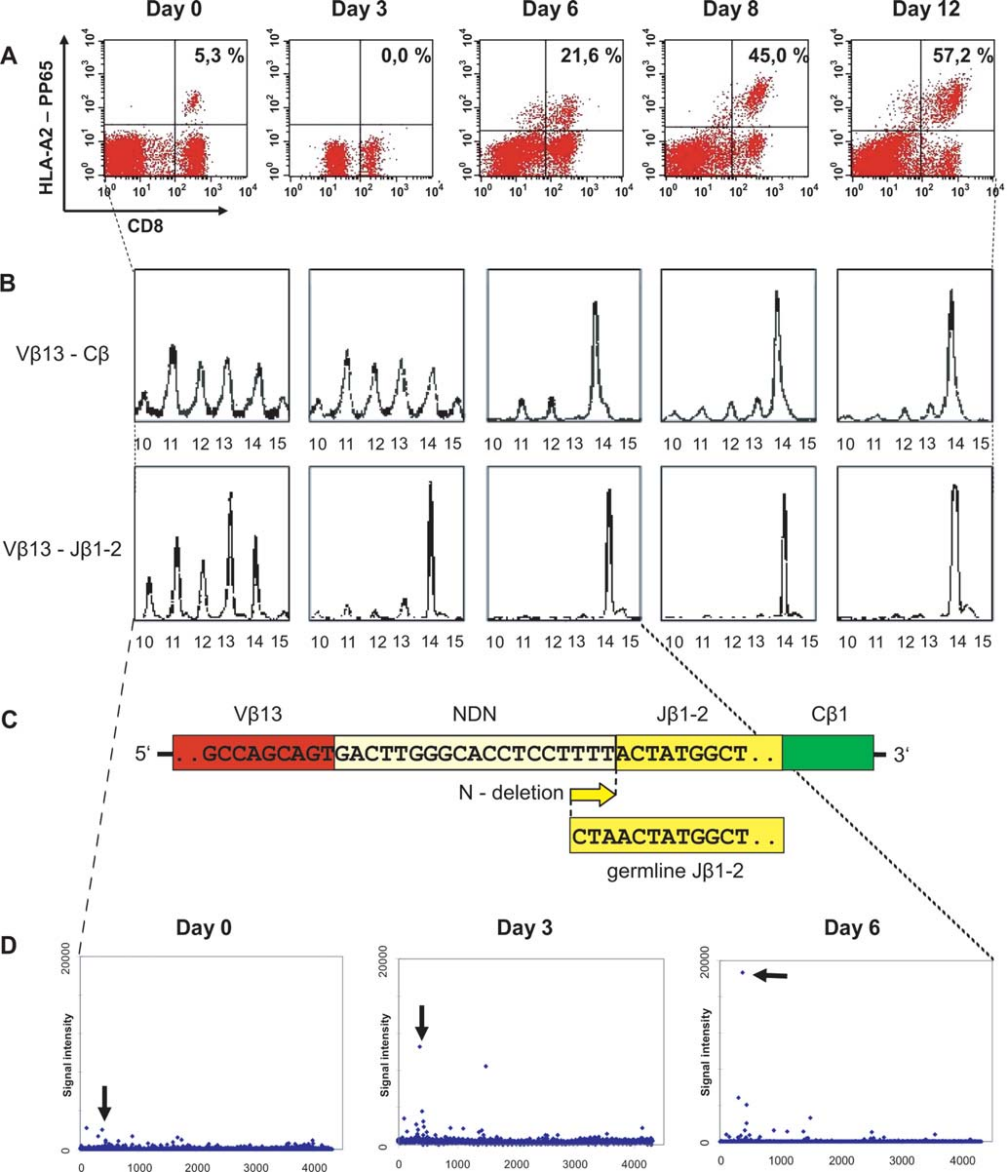
Clonal expansion of T-cells from a CMV^+^ donor after antigen specific stimulation. **(panel A)** The fraction of antigen specific T-cell clones determined with NLVPMVATV-loaded tetramers. **(panel B)** Spectratyping of unsorted Vβ13^+^ and Vβ13^+^/Jβ1-2^+^ fraction. **(panel C)** CDR3 sequence of the clone identified compared to the Jβ1-2 germline sequence. **(panel D)** T-array of unsorted Vβ13^+^/Jβ1-2^+^ fraction. The annealer oligonucleotide used in this T-array experiment had the sequence ACTATGGCTACACCTTCGGTT, allowing detection of rearrangements with 3 or less nucleotides deleted from the Jβ1-2 germline. The arrow indicates sequence CCTTTT, the first nucleotides of the NDNβ region of the dominant T-cell clone that was identified in this screen.

To test whether the T-array protocol would allow identification of T-cell clones that expand upon antigen activation an in vitro stimulation experiment was performed. Human peripheral blood cells from a healthy HLA-A2^+^ donor latently infected with the β herpes virus CMV were isolated and stimulated with the CMV-peptide NLVPMVATV. This 9-amino acid motif from the viral structural protein pp65 dominates the cytotoxic T-lymphocyte response against CMV [15]. In HLA-A2^+^ individuals, the CD8^+^ response to NLVPMVATV is Vβ-restricted, in particular for but not limited to Vβ13^+^ T-cells [16], which was in agreement with spectratyping analysis of our donor (data not shown). FACS analysis, using HLA-A2-NLV tetramer staining showed that the fraction of antigen specific T cells in the cytotoxic T-cell pool increased after stimulation with NLV peptide (**Fig. 4A**). Before stimulation (Day 0) a fraction of ∼5% of the CD8^+^ cells was tetramer positive confirming CMV latency. Three days after peptide stimulation CMV-reactive T-cells were not detectable by FACS using tetramers, which can be attributed to TCR internalization after MHC/peptide recognition [17]. During the next 10 days, the fraction of tetramer-positive cells slowly increased to ∼60%. From day 6, spectratyping analysis revealed that the Vβ13^+^ compartment became restricted to a CDR3 length correlating to 14 amino acids (**Fig. 4B**), suggesting that either a single T-cell clone or only a limited number of clones in the Vβ13 compartment had expanded.

While Vβ-13/Cβ-spectratyping (**Fig. 4B**) or tetramer analysis (**Fig. 4A**) only detected antigen-specific clones for CMV at day 6, this clone was detected at day 3 using a more specific Vβ-13/Jβ-1-2 spectratyping approach. The T-array, which was performed on non-sorted T-cells, identified the CMV-specific T-cell clone characterised by the NDN sequence CCTTTT already at day 0 (**Fig. 4C**). To exclude aspecific effects of the primer and annealer sequences used a T-array experiment was performed using the same primers and annealer oligonucleotide on a different sample; in this case no signal above background was observed at the CCTTTT hexamer (data not shown). Further validation of the CCTTTT hexamer sequence was acquired using extensive cloning and sequencing (See below).

Hexamer sequences other than CCTTTT gave signals above background intensity (**Table S3**, **Fig. 4C**). Of the top 100 signals 19 had a 3′ CTA-end, identical to the terminal germline sequence of the Jβ-1-2 gene segment. These sequences derive from TCRs that have no nucleotides deleted from the germline Jβ-1-2 gene (**Fig. S2**), and therefore give 5′-(NNN)CTA-3′ signals. Similarly, sequences that have a (NNNN)TA-end (34 out of 100) or (NNNNN)A-end (62 out of 100) derive from TCRs encoding the germline Jβ-1-2 gene with only one resp. two terminal nucleotide deleted. Indeed, these signals derive from such TCR sequences as shown by complete sequencing of these TCRβ's (see also below). They can all be identified based on the terminal germline nucleotide (here “A”) in the hexamer sequence.

### Validating T-array data by sequencing of multiple T-cell clones

To test whether the T-array signal matches the frequency of these T-cell clones as estimated by repetitive cloning and sequencing, we sequenced Vβ13^+^/Jβ1-2^+^ TCRs from three samples of the experiment shown in Figure 4 (**Table S4**). Out of 52 clones sequenced from the Day 0 sample, 27 were found to have 3 or less nucleotides deleted from the Jβ-1-2 gene, and can therefore be detected in the T-array shown in Figure 4. Fourteen of these sequences were unique. The two clones that were found at high frequency (7/52) gave the strongest signals on the T-array (hexamer sequences CCTTTT and GGACCG). One clone which was detected at lower frequency (CAGCTA, frequency 2/52) also gave a T-array signal well above background. Eleven clones were detected with a frequency of only 1 out of 52. Three of these gave T array signals above background (**Table S5**). Eight clones gave signals similar to background, suggesting that the concentration of these clones in the blood sample is below the detection limit of the T-array. The clonal frequencies measured at day 3 were in agreement with the expansion measured by the T-array, showing that the T-array protocol quantitatively detects clonal expansion.

### Application of T-array protocol for *in vivo* detection

**Figure 5:**
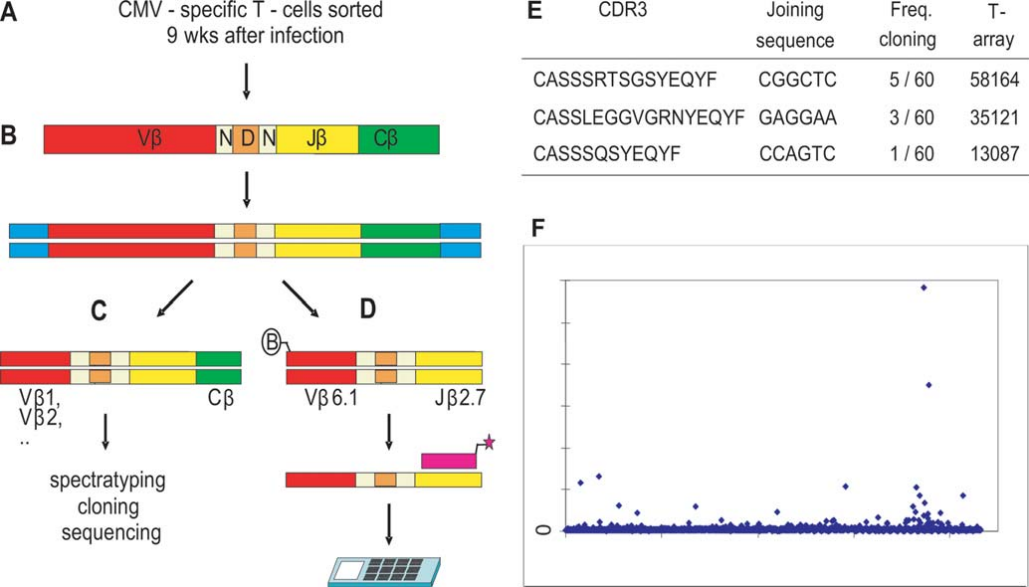
T-array analysis of CMV-specific cells *in vivo.* **(A)** Peripheral blood was drawn 9 weeks after primary CMV infection of a CMV-seronegative renal transplant recipient, and sorted for CMV+ IFNγ-producing T-cells. **(B)** cDNA was amplified using anchored PCR (*ref. 20*). **(C)** Vβ's were analyzed by spectratyping and 11 Vβ-families were cloned and sequenced (*ref. 18*). **(D)** The most prominent Vβ/Jβ combination as identified by (C), i.e. the Vβ6.1/Jβ2.7+pool, was amplified by Vβ6.1-and Jβ2.7-primers and loaded on a hexamer array. **(E)** CDR3 regions, clonal frequencies, and joining sequences of the identified Vβ6.1/Jβ2.7+T-cell clones. **(F)** Vβ6.1/Jβ2.7-specific T-array with the annealer CTACGAGCAGTACTTCGGG, which matches the germline Jβ-2-7 sequence with 3 nucleotides deleted.

To test the applicability of the T-array protocol for detection of clones *in vivo,* we analyzed a well-characterized sample of FACS sorted, CMV-specific, IFNγ-secreting CD4^+^ T-cells from a renal transplant recipient 9 weeks after primary CMV infection, at the peak of viral load [18]. This sample of 11,600 sorted CMV-specific T-cells was pre-amplified by anchored PCR [19], [20], which was used here as pre- amplification step to generate sufficient cDNA from a relatively small amount of RNA (**Fig. 5A–B**). Spectratyping indicated a relatively broad repertoire [18]. Within the repertoire 11 Vβ families were extensively analyzed by cloning and sequencing [18]. In the Vβ6.1 pool, 60 clones were sequenced, revealing 12 unique sequences of which 4 were Jβ2.7^+^. A T-array was performed to screen the Vβ6.1- Jβ2.7 subpopulation with an annealer oligonucleotide that detects Jβ2.7 sequences with 3 or less nucleotides deleted from the Jβ2.7 gene (**Fig. 5F**). All 3 clones that meet these criteria were picked up with the T-array (**Fig. 5E**, **Table S5**). In addition, the T-array signal matched the clonal frequency of the T-cell clones identified. The clone with hexamer CGGCTC which was picked up in 5 out of 60 sequences gave the strongest signal, followed by clone GAGGAA (3 out of 60), and clone CCAGTC (1 out of 60), respectively. These data show that the T-array can detect *in vivo* expanded T-cell clones in a quantitative way.

## Discussion

The diverse repertoire of TCR rearrangements can potentially be analyzed using microarrays, which have a high capacity to differentiate and monitor many unique DNA rearrangements in parallel. However, the size of the TCRβ repertoire at the DNA level is too large for full TCR repertoire analysis at single-clone resolution on a single microarray. The αβ receptor diversity is estimated at 10^15^ to 10^18^ rearrangements [1], [21], which is formed for a relatively large part by the β chain. Within one individual, however, the size of the β chain repertoire is much more limited (10^6^) [2], and microarrays could produce sufficient resolution to distinguish single T cell clones in the repertoire of one individual. Here, we use universal microarrays for this concept and show that this is feasible. In the design presented, T-arrays tag individual clones based on the sequence information in the NDN-J or NDN-V junction. The tag for each clone consists of the J- or V-family used, the number of terminal nucleotides deleted from this J- or V-gene segment and the first six nucleotides of the NDN region. This design creates tags that are specific for one in more than a million clones, which in theory allows single-clone analysis of the complete TCRβ repertoire on high-density microarrays.

The validity of the T-array protocol was shown in several experiments. Firstly, the PCR fragments derived from the TCR of Jurkat cells were selectively ligated to hexamer oligonucleotides complementary to its NDN sequence both in solution and using hexamer arrays (**Fig. 2**). Second, the protocol allowed early, highly specific identification of an expanding T cell clone after in vitro stimulation with CMV-peptide (**Fig. 4**). Third, T-cell clones from blood taken form CMV-infected individuals that were identified using T-arrays, were also detected by multiple cloning and sequencing (**Table S4**). Lastly, in FACS-sorted CMV-specific, IFNγ-secreting CD4+ T cells from a renal transplant patient 9 weeks after CMV infection T-arrays detected the dominant Vβ6.1^+^/Jβ2.7^+^ clones identified earlier by extensive cloning and sequencing (**Fig. 5**).

**Figure 6:**
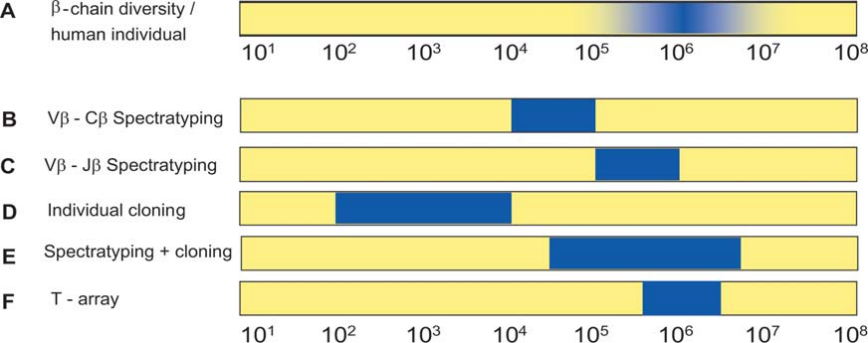
Comparison of the diversity of the human TCRb repertoire and the sensitivity of various methods for repertoire analysis. **(A)** The human T-cell repertoire contains an estimated 10^6^ rearrangements per individual (*ref. 2*). **(B)** The sensitivity of Vβ/Cβ immunoscope is approximately 2 in 10^4^ (*ref. 15 and* *Fig. 3A*). **(C)** Based on the number of Jβ primers, it is estimated that Vβ/Jβ immunoscope is 12-fold more sensitive than Vβ-Cβ immunoscope. **(D,E)** The sensitivity by which TCR rearrangements are picked by indivual cloning, depends on the number of clones sequenced (*ref. 5–9*). **(F)** The sensitivity of the T-array is approximately 1 in 10^6^ rearrangements (*Fig. 3C*).

The sensitivity of the protocol was determined after mixing a Jurkat T-cell clone in a background of peripheral blood CD4+ T-cells in a range of dilutions. The data show that the Jurkat TCR rearrangement was detected in a ratio of at least 1 in 10^6^ (**Fig. 3**). This is 2 logs more sensitive than Vβ-Cβ spectratyping [4], [15], which can detect a T-cell clone in 1 in 10^4^. Vβ-Jβ spectratyping, an alternative approach which is not widely used, is theoretically 12-fold higher than that of Vβ spectratyping and therefore 10-fold less sensitive than the T-array approach. The superior sensitivity of the T array was confirmed by detection of a CMV-specific T-cell clone which was identified in the unstimulated population of circulating T cells obtained directly from a CMV-infected donor (**Fig. 3**). This clone was only detected after 3 days of antigen stimulation by Vβ-Jβ spectratyping and after 6 days by Vβ-Cβ spectratyping (**Fig. 4**). Thus T-arrays make highly sensitive detection and tracking of T cells possible. Figure 6 illustrates the sensitivity of various methods for the analysis of the TCRβ repertoire.

In addition, the protocol allowed quantitative monitoring of T cell clones. With decreasing numbers of Jurkat cells in a CD4 background the signal clearly decreased (**Fig. 3**). The increasing frequency of the CMV-specific clone in the in vitro experiment as evidenced by tetramer staining, and by spectratyping, was also reflected in signal intensities on the arrays (**Fig. 4**). Likewise, the observed clonal frequencies of the CMV-specific clones in the in vivo experiment (**Table S4**) were quantitatively reflected in the T-array data.

Although the ligation reaction is highly specific for the correct hexamer sequence, ligation mismatches did occur. However, in every instance true positives gave the strongest signal, even in complex mixtures. Figure 2G supports previous data [14], [22] which show that mismatches occur mainly in the two nucleotide positions opposite of the site of ligation. Algorithms, based on known ligation patterns [14], [22], have been developed that identify false positives and reduce the loss of resolution when complex mixtures such as full-genome transcripts are analyzed on hexamer arrays [14]. Such algorithms may help to minimize the effect of cross ligations on the resolution of T-arrays and help to detect less frequent clones.

The technology described here can be applied to monitor a small selection of the TCRβ repertoire quantitatively, and to track a subset of T-cell clones sensitively and quantitatively. While the combination of spectratyping, cloning and sequencing may take several weeks, the T-array method takes only a single day including scanning and quantification. Furthermore it is sensitive, and allows monitoring of growth kinetics at the clonal level. This rapid and sensitive method may find applications in the study of the relation between clonal expansion of T cells and autoimmune phenomena, e.g. responses to immunotherapy, retrospectively and prospectively. Recurrence of autoimmune disease could be predicted in the case of previously identified clones [23], or the fate of T-cells in adoptive therapy against cancer [24] could be monitored at single-clone level.

One of the prospects of this technology is that it could possibly be developed into a tool that screens the complete TCRβ repertoire on a single array. The format presented here screens only 1/23 · 1/144 = 0.03% of the repertoire (**Table S2C**). Recently, we successfully explored the feasibility of a protocol in which T-array analysis is preceded by simultaneous amplification of all Vβ families in one PCR reaction using anchored PCR as described earlier [19], [20] (data not shown). The resulting 144 arrays can then be housed in a high-density matrix of multiple arrays that can be individually loaded. Such matrices have recently become available [25]. Rapid, quantitative and sensitive full repertoire screening would have significant impact in immunological research and on the development of immunotherapeutics. Identical arrays might be built for the analysis of the TCRα, -γ and -δ repertoires and of the B-cell receptor repertoire in humans and other species.

In conclusion, here we show proof of concept of an approach to sensitively monitor changes in the frequency of unique TCR rearrangements using microarrays. The protocol is rapid and universal for the detection of all T- and B-cell receptor rearrangements. We propose that this technology will be useful for monitoring of clonal T- and B-cell expansions for many applications in medical research.

## Materials and Methods

### Analysis of CDR3 sequences from public database

TCR β-CDR3 mRNA sequences of human T-cell clones were collected from the public database of NCBI at NIH. Vβ-, Jβ-, Dβ-segments were identified using the V-QUEST algorithm from the international ImMunoGeneTics information system [13]. A number of 50 sequences were validated manually, and assignment errors were identified only for N-deletions larger than 8 nucleotides. To exclude other assignment errors all CDR3β sequences with N-deletions larger than 7 nucleotides were therefore assigned manually.

### Cells and flow cytometry

Jurkat cell line clone E6-1 (ATCC, Manassas, VA) was grown in DMEM culture medium (Sigma-Aldrich, St. Louis, MO) supplemented with 5% FCS. Human peripheral blood mononuclear cells (PBMC) (Figure 3) were isolated from buffy coats of healthy blood donors by density centrifugation with Ficoll-Isopaque (Pharmacia Biotech, Uppsala, S). Informed consent was obtained from blood donors. CD4^+^ T cells were isolated by using anti-CD4 microbeads (Miltenyi Biotec, Bergisch Gladbach, D), followed by positive selection with the VarioMACS (Miltenyi Biotec) according to the manufacturer's protocol. The purity of the CD4+ cells isolated was measured using anti-CD4 PerCP-conjugated antibodies (BD Biosciences, San Jose, CA).

Thawed PBMCs (Figure 4) were resuspended in IMDM (BioWhittaker, Verviers, Belgium), containing 10% FCS and antibiotics (100 U/ml sodium penicillin G and 100 µg/ml streptomycin sulfate). Cells were washed in PBS containing 0.01% (w/v) NaN_3_ and 0.5% (w/v) BSA (PBA). A total of 250,000 PBMCs were incubated with an appropriate concentration of tetrameric complexes in a small volume for 10 min at 4°C. Subsequently, fluorescently labelled conjugated mAbs (concentrations according to manufacturer's instructions) were added and incubated for 30 min at 4°C. For analysis of expression of surface markers, the following Abs were used: the allophycocyanin-conjugated HLA-A2 tetramer loaded with the CMV pp65-derived NLVPMVATV peptide [15], and anti-CD8 PerCP–conjugated antibodies (BD Biosciences, San Jose, CA).

CMV-specific IFN-γ-producing CD4+ cells from a renal transplant recipient were isolated using IFNγ Secretion Assay Detection Kit (PE) (Miltenyi Biotec, Amsterdam, The Netherlands) according to the manufacturer's conditions. At the moment of peak viral load, 9 weeks after transplantation, PBMCs were isolated and stimulated for 16 hours with CMV antigen (10 µl/ml) and incubated with IFNγ Catch Reagent for 5 minutes at 4°C, incubated with IFNγ Detection Antibody (PE), CD4 APC (BD Pharmingen, San Diego, USA) and sorted using FACsARIA (BD). The patient had given written informed consent, and the local medical ethics committee had approved the study.

### Expansion of virus-specific autologous cytotoxic T-lymphocytes

PBMCs from a CMV seropositive, HLA-A2^+^ healthy volunteer donor were used for expansion of CMV specific CD8^+^ cells. Informed consent was obtained from the blood donor. PBMCs were stimulated in IMDM supplemented with 10% human pool serum, antibiotics, and 2-ME with CMVpp65-A2 peptide NLVPMVATV (1.25 µg/ml) and IL-2 (50 U/ml Biotest, Dreieich, D) in 24-well plates. After one week, cells were restimulated on a weekly basis with irradiated (30 Gy) CMV-pp65-A2 peptide loaded EBV transformed cell-lines expressing HLA-A2^+^ (5×10^4^ cells/ml) in the presence of IL-2.

### Cloning and sequencing

Vβ PCR products were purified and ligated into pGEM-T Easy Vector (Promega, Madison, WI) and cloned by transformation of competent DH5α *E. coli*. Selected colonies were amplified by PCR using M13 primers (Invitrogen - Life Technologies, Breda, NL) and then sequenced on the ABI Prism 3730 DNA automatic sequencer (Applied Biosystem, Foster City, CA) using the dye terminator cycle sequencing chemistry (v1.1) (Perkin Elmer, Foster City, CA). Clones that did not yield a PCR product using direct colony-PCR, were cultured in LB medium, plasmid DNA was purified using the Wizard Plus Minipreps DNA purification system (Promega, Madison, WI), and plasmids were sequenced similarly as described above.

### PCR and Spectratyping analysis

RNA was isolated using the GenElute Mammalian Total RNA Kit (Sigma-Aldrich, Zwijndrecht, NL). For experiments shown in Figures 2, 3 and 4, cDNA was synthesized using Superscript RT II and oligo-dT primers (Sigma-Aldrich) according to the manufacturer's protocol (InVitrogen - Life Technologies, Breda, NL). For experiments shown in Figure 5, cDNA was synthesized using the Super Smart™ and the Smart™ cDNA synthesis kit (Clontech, Mountain View, CA), respectively. PCR was performed with TCR Vβ primers [26] in combination with a TCR Cβ primer, labelled with fluorescent dye fluorophore fluorescamine (FAM). Each amplification reaction was performed with 4 μl cDNA in the presence of 25 pmol 5′ sense TCR Vβ primer, 25 pmol 3′ antisense TCR Cβ primer, 0.5 mM MgCl_2_, 0.5 mM dNTP, 10 mM Tris–HCl (pH 8.4), 50 mM KCl, 4 mM KCl, 2.5 units AmpliTaq DNA polymerase (Perkin Elmer/Roche Molecular Systems Inc., Branchburg, NJ) in a total volume of 40 µl. PCR cycles were performed in a T1 Thermocycler (Biometra, Goettingen, D). The FAM-labelled PCR products were run on the ABI Prism 3100 Genetic Analyzer capillary system (Applied Biosystem, Foster City, CA) using POP6 as separation matrix, filter set D for the detection of fluorescent signals, and ROX500 as internal size standard. Genescan Software (Applied Biosystem, Foster City, CA) was used for size determination and quantification.

### T-array protocol

After cDNA synthesis (1C1), the T-array protocol follows PCR amplification (1C2), isolation of polyclonal, single strands (1C3), hybridization of annealer oligonucleotides (1C4), and ligation, washing, scanning, and quantification of hexamer arrays (1C5–C7).

#### PCR amplification (Fig. 1C2)

Biotinylated PCR products were obtained using sense biotinylated Vβ primers against reverse, antisense Cβ or Jβ primers (PCR conditions as described above). For the analysis of CMV-cells *in vivo* (**Fig. 5**), cDNA was synthesized using the smart PCR cDNA synthesis kit (Clontech, Mountain View, CA).

#### Isolation of single strands (Fig. 1C3)

1.0 mg streptavidin-coated magnetic beads (M-280 Dynabeads, Dynal Biotech, Oslo, N) were washed twice in B&W buffer (Dynal Biotech, Oslo, N) and biotinylated PCR products were linked to the magnetic beads according to the suppliers protocol. Non-bound DNA and nucleotides were removed by washing in 1x and subsequently 0.4× B&W buffer. The non-biotinylated single strands were released by 10 minutes incubation in 0.15 N NaOH. After magnetic separation, supernatant containing the non-biotinylated single strands was pH neutralized using neutralization buffer (0.75 HCl, 0.125 M Tris, 16.7 mM MgCl_2_, 1.67 mM DTT).

#### Hybridization of annealer oligonucleotides (Fig. 1C4)

Single strands were then incubated with 1 pmol Cy5-labeled, 5′ phosphorylated annealer oligonucleotide (Biolegio, Malden, NL) at a starting temperature of 90°C. The heated water bath was (passively) cooled to ambient temperature. Sequences of used annealer oligonucleotides are CTAACTATGGC-TACACCTTCGGTTT (Fig. 2, 3), AAACTGCTGGCACAGAAGTACACTT (Fig 2d,e), ACTATGGCTACACCTTCGGTT (Fig. 4) and CTACGAGCAGTACTTCGGG (Fig. 5).

#### Ligation, washing, scanning, and quantification (Fig. 1C5–C7)

Ligation on arrays (Accessarray, Expresson Biosystems, Roslin, UK) was performed at 30°C in a volume of 125 µl in 1× BSA (NEB, Ipswich, MA), 25 µl 5× DNA Ligase buffer, 12 units T4 DNA ligase. After ligation slides were washed in 0.1% SDS at 90°C, ddH2O at RT, and dried by 500× *g* centrifugation for 3 minutes. Ligated arrays were scanned with a GSI Lumonics ScanArray 5000 (Perkin-Elmer Life Sciences, Boston, MA). Spot intensities were quantified using with ArrayVision 6.0 software (Image Research, St. Catharines, Ontario, CDN).

T-array data in a format according to the MIAME guidelines checklist www.mged.org/Workgroups/MIAME/miame_checklist.html are available on request.

### Ligation in *solution*

For experiments shown in **Fig. 2B–E**, ligation was performed *in solution* with single hexamer oligonucleotides. 1 pmol of hexamers, 4 units of T4 DNA ligase and 2 µl 5× DNA Ligase buffer (In vitrogen - Life Technologies, Breda, NL) and template/annealer complex were added in a total volume of 10 µl and incubated for 45 minutes at 16°C, followed by a 10 minutes denaturation step at 65°C. Ligation products were analyzed on the ABI Prism 3100 Genetic Analyzer capillary system and Genescan software as described above.

## Supporting Information

Figure S1 Semi-quantitative PCR Jurkat cells and CD4+ cells.
(0.06 MB DOC)

Figure S2 Germ line signals in T-array.
(0.05 MB DOC)

Table S1 N-deletion in 192 TCRβ sequences from public databases.
(0.51 MB DOC)

Table S2 Maximum resolution for spectratyping and T-array protocol.
(0.06 MB DOC)

Table S3 Signal intensity T arrays.
(0.12 MB DOC)

Table S4 Comparing T-array signals to results from cloning and sequencing in the ex vivo CMV-stimulation experiment.
(0.29 MB DOC)

Table S5 T-array analysis of CMV-specific cells *in vivo*.
(0.07 MB DOC)
