# Supplementary material for: Monitoring the T-Cell Receptor Repertoire at Single-Clone Resolution
Source: PLoS One. 2006 Dec 20;1(1):e55. doi: 10.1371/journal.pone.0000055 (PMC1762342; doi:10.1371/journal.pone.0000055)
Supplement: Figure S1 — Semi-quantitative PCR Jurkat cells and CD4+ cells. (0.06 MB DOC) [file pone.0000055.s001.doc]

**FIGURE S1**. **Semi-quantitative PCR** **Jurkat cells and CD4+ cells.** Jurkat cells and peripheral blood CD4+ cells were counted and diluted to 10^6^ and 2*10^6^ cells/ml. cDNA was prepared and amplified with primers specific for β-actine and TCRβ-constant region in 18 PCR cycles. The PCR shows TCR transcripts in Jurkat cells are not more abundant than in CD4+ cells obtained from a healthy blood donor.


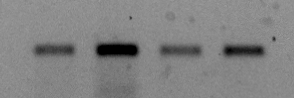

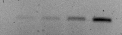


TCR

β

-

C

β

-

Actin

10

6

2*10

6

10

6

2*10

6

Number

of cells

Cell type

Jurkat PB CD4s
