# Supplementary material for: Monitoring the T-Cell Receptor Repertoire at Single-Clone Resolution
Source: PLoS One. 2006 Dec 20;1(1):e55. doi: 10.1371/journal.pone.0000055 (PMC1762342; doi:10.1371/journal.pone.0000055)
Supplement: Figure S2 — Germ line signals in T-array. (0.05 MB DOC) [file pone.0000055.s002.doc]

**FIGURE S2. Germ line signals in T-array**. In the T-array protocol, annealer nucleotides are designed to anneal to Jβ genes of which a specific number of nucleotides are deleted from the germ line sequence (**Fig. 1C**). For example, a Jβ annealer (hatched purple in figure) specific for clones of which 3 nucleotides have been deleted gives a ligation event for T-cell clones that meet this criterium (**example A**). However, T-cell clones with less than 3 nucleotides deleted, for example with no germline deletions (**example B**) also give a ligation event with the same annealer. On the T-array, these are recognized as germ line signals (CTA in example B).

**654321**

**CCTTTT**

N D N

J

β

C

β

**654321**

**TGGCTA**

**A**

**B**

**3 nucleotides deleted**

**from J**

**β**

**germ line DNA**

**no nucleotides deleted**

**from J**

**β**

**germ line DNA**

V

β

N D N

J

β

C

β

V

β
