# Supplementary material for: Monitoring the T-Cell Receptor Repertoire at Single-Clone Resolution
Source: PLoS One. 2006 Dec 20;1(1):e55. doi: 10.1371/journal.pone.0000055 (PMC1762342; doi:10.1371/journal.pone.0000055)
Supplement: Table S1 — N-deletion in 192 TCRβ sequences from public databases. (0.51 MB DOC) [file pone.0000055.s003.doc]

**TABLE S1. N-deletion in 192 TCRβ sequences from public databases.** The Table shows the NCBI accession number, 5’-end sequence of Vβ DNA, 3’-end sequence of Jβ cDNA, and the number of deleted nucleotides from the germ line sequences. Nomenclature according to Rowen et al.^1^

| **Access nr** | **V-family** | **V-sequence** | **N-del V** | **J-family** | **J-sequence** | **N-del J** |
| --- | --- | --- | --- | --- | --- | --- |
| AY082732 | TRBV2*01 | TGTGCCAGC........ | 8 | J1-1*01 | ...ACACTGAAGCTTTCTTT | 3 |
| AY006295 | TRBV4*01 | TCTGCGCCAGCAG...... | 5 | J1-1*01 | .......TGAAGCTTTCTTT | 7 |
| AF043764 | TRBV12-4*02 | TGTGCCAGCAGTTTA | 0 | J1-1*01 | ...........GCTTTCTTT | 11 |
| AF043770 | TRBV27*01 | TGTGCCAGCAGTTT... | 3 | J1-1*01 | .........AAGCTTTCTTT | 9 |
| AF043798 | TRBV28*01 | TGTGCCAGCAGTT.... | 4 | J1-1*01 | ....CACTGAAGCTTTCTTT | 4 |
| AF043803 | TRBV12-4*02 | TGTGCCAGCAGTTT. | 1 | J1-1*01 | ........GAAGCTTTCTTT | 8 |
| AF043817 | TRBV27*01 | TGTGCCAGCAGTTTA.. | 2 | J1-1*01 | ....CACTGAAGCTTTCTTT | 4 |
| AF043818 | TRBV27*01 | TGTGCCAGCAG...... | 6 | J1-1*01 | .....ACTGAAGCTTTCTTT | 5 |
| AF043840 | TRBV12-4*02 | TGTGCCAGCAGT... | 3 | J1-1*01 | ..AACACTGAAGCTTTCTTT | 2 |
| AF043863 | TRBV19*03 | TGTGCCAGTAG. | 1 | J1-1*01 | .GAACACTGAAGCTTTCTTT | 1 |
| AF043864 | TRBV19*03 | TGTGCCAGTAG. | 1 | J1-1*01 | .GAACACTGAAGCTTTCTTT | 1 |
| AF043865 | TRBV19*03 | TGTGCCAGTAG. | 1 | J1-1*01 | ......CTGAAGCTTTCTTT | 6 |
| AF043866 | TRBV19*03 | TGTGCCAGTA.. | 2 | J1-1*01 | TGAACACTGAAGCTTTCTTT | 0 |
| AF079014 | TRBV28*01 | TGTGCCAGCAGT..... | 5 | J1-1*01 | ...ACACTGAAGCTTTCTTT | 3 |
| M13849 | TRBV5-1*01 | TGCGCCAGCAGCTT.. | 2 | J1-1*01 | ............CTTTCTTT | 12 |
| S63248 | TRBV28*01 | TGTGCCAGCAGTT.... | 4 | J1-1*01 | .....ACTGAAGCTTTCTTT | 5 |
| U34971 | TRBV4-1*01 | TGCGCCAGCAGCCAAGA | 0 | J1-1*01 | TGAACACTGAAGCTTTCTTT | 0 |
| Z77833 | TRBV5-1*01 | TGCGCCA......... | 9 | J1-1*01 | ........GAAGCTTTCTTT | 8 |
| AF043766 | TRBV12-4*02 | TGTGCCAGCAGTT.. | 2 | J1-2*01 | .......TGGCTACACCTTC | 7 |
| AF043771 | TRBV27*01 | TGTGCCAGCAGTTTATC | 0 | J1-2*01 | ....CTATGGCTACACCTTC | 4 |
| AF043772 | TRBV27*01 | TGTGCCAGCAG...... | 6 | J1-2*01 | CTAACTATGGCTACACCTTC | 0 |
| AF043773 | TRBV27*01 | TGTGCCAGCAGT..... | 5 | J1-2*01 | ..AACTATGGCTACACCTTC | 2 |
| AF043774 | TRBV27*01 | TGTGCCAGCAGTTTA.. | 2 | J1-2*01 | ....CTATGGCTACACCTTC | 4 |
| AF043775 | TRBV27*01 | TGTGCCAGCAGTTTA.. | 2 | J1-2*01 | ....CTATGGCTACACCTTC | 4 |
| AF043776 | TRBV27*01 | TGTGCCAGCAGTTT... | 3 | J1-2*01 | .....TATGGCTACACCTTC | 5 |
| AF043777 | TRBV27*01 | TGTGCCAGCAGTTT... | 3 | J1-2*01 | ....CTATGGCTACACCTTC | 4 |
| AF043778 | TRBV27*01 | TGTGCCAGCAGTTTA.. | 2 | J1-2*01 | .....TATGGCTACACCTTC | 5 |
| AF043779 | TRBV27*01 | TGTGCCAGCAGT..... | 5 | J1-2*01 | ....CTATGGCTACACCTTC | 4 |
| AF043780 | TRBV27*01 | TGTGCCAGCAG...... | 6 | J1-2*01 | .....TATGGCTACACCTTC | 5 |
| AF043781 | TRBV27*01 | TGTGCCAGCAG...... | 6 | J1-2*01 | .....TATGGCTACACCTTC | 5 |
| AF043782 | TRBV27*01 | TGTGCCAGCAGTT.... | 4 | J1-2*01 | ....CTATGGCTACACCTTC | 4 |
| AF043791 | TRBV19*03 | TGTGCCAGT... | 3 | J1-2*01 | CTAACTATGGCTACACCTTC | 0 |
| AF043799 | TRBV28*01 | TGTGCCAGCAG...... | 6 | J1-2*01 | ......ATGGCTACACCTTC | 6 |
| AF043841 | TRBV12-4*02 | TGTGCCAGCAGT... | 3 | J1-2*01 | ....CTATGGCTACACCTTC | 4 |
| AF043885 | TRBV27*01 | TGTGCCAGCAGTTTA.. | 2 | J1-2*01 | ....CTATGGCTACACCTTC | 4 |
| AF043887 | TRBV27*01 | TGTGCCAGCAGTTT... | 3 | J1-2*01 | ....CTATGGCTACACCTTC | 4 |
| AF043891 | TRBV27*01 | TGTGCCAGCAGTTTA.. | 2 | J1-2*01 | .....TATGGCTACACCTTC | 5 |
| AF043893 | TRBV27*01 | TGTGCCAGCAGT..... | 5 | J1-2*01 | ..AACTATGGCTACACCTTC | 2 |
| AF043895 | TRBV27*01 | TGTGCCAGCAGT..... | 5 | J1-2*01 | ..AACTATGGCTACACCTTC | 2 |
| AJ549940 | TRBV29-1*01 | TGCAGCGTTG.... | 4 | J1-2*01 | CTAACTATGGCTACACCTTC | 0 |
| AJ549944 | TRBV20-1*02 | TGCAGTGCT | 0 | J1-2*01 | ....CTATGGCTACACCTTC | 4 |
| AJ549947 | TRBV12-3*01 | TGTGCCAGCAGT..... | 5 | J1-2*01 | ...ACTATGGCTACACCTTC | 3 |
| K02779 | TRBV12-4*01 | TGTGCCAGCAGTTT... | 3 | J1-2*01 | CTAACTATGGCTACACCTTC | 0 |
| L34737 | TRBV28*01 | TGTGCCAGCAG...... | 6 | J1-2*01 | ...ACTATGGCTACACCTTC | 3 |
| M86362 | TRBV5-5*02 | TGTGCCAGCAGC | 0 | J1-2*01 | .....TATGGCTACACCTTC | 5 |
| Z81025 | TRBV29-1*01 | TGCAGCGT...... | 6 | J1-2*01 | ....CTATGGCTACACCTTC | 4 |
| X92784 | TRBV10-3*01 | TGTGCCATCAG...... | 6 | J1-2*01 | .TAACTATGGCTACACCTTC | 1 |
| AJ405776 | TRBV10-3*01 | TGTGCCATCAGTGAG.. | 2 | J1-2*01 | ....CTATGGCTACACCTTC | 4 |
| AF079028 | TRBV28*01 | TGTGCCAGCAGTTT... | 3 | J1-2*01 | ............ACACCTTC | 12 |
| AF043753 | TRBV28*01 | TGTGCCAGCA....... | 7 | J1-3*01 | CTCTGGAAACACCATATATTTT | 0 |
| AF043754 | TRBV28*01 | TGTGCCAGCA....... | 7 | J1-3*01 | .........CACCATATATTTT | 9 |
| AF043783 | TRBV27*01 | TGTGCCAGCAGT..... | 5 | J1-3*01 | CTCTGGAAACACCATATATTTT | 0 |
| AF043784 | TRBV27*01 | TGTGCCAGCAG...... | 6 | J1-3*01 | CTCTGGAAACACCATATATTTT | 0 |
| AF043804 | TRBV12-4*02 | TGTGCCAGCAG.... | 4 | J1-3*01 | ....GGAAACACCATATATTTT | 4 |
| AF043805 | TRBV12-4*02 | TGTGCCAGCAG.... | 4 | J1-3*01 | ...TGGAAACACCATATATTTT | 3 |
| AF043842 | TRBV12-4*02 | TGTGCCAGCAGT... | 3 | J1-3*01 | ....GGAAACACCATATATTTT | 4 |
| S63249 | TRBV27*01 | TGTGCCAGCAGT..... | 5 | J1-3*01 | CTCTGGAAACACCATATATTTT | 0 |
| AJ405806 | TRBV4*01 | TGCGCCAGCAGCCAAGA | 0 | J1-3*01 | ....GGAAACACCATATATTTT | 4 |
| X04935 | TRBV10-3*01 | TGTGCCAT......... | 9 | J1-3*01 | CTCTGGAAACACCATATATTTT | 0 |
| S67402 | TRBV4*01 | TCTGTGCCTGCAGCC.... | 4 | J1-4*01 | ......ATGAAAAACTGTTTTTT | 6 |
| AF043800 | TRBV28*01 | TGTGCC........... | 11 | J1-4*01 | ........GAAAAACTGTTTTTT | 8 |
| X92780 | TRBV10-3*01 | TGTGCCATCAGTG.... | 4 | 1-4*01 | ....TAATGAAAAACTGTTTTTT | 4 |
| AF043767 | TRBV12-4*02 | TGTGCCAGCAG.... | 4 | J1-5*01 | ....AATCAGCCCCAGCATTTT | 4 |
| AF043792 | TRBV19*03 | TGTGCCAGTAG. | 1 | J1-5*01 | ...CAATCAGCCCCAGCATTTT | 3 |
| AF043806 | TRBV12-4*02 | TGTGCCAGCAG.... | 4 | J1-5*01 | TAGCAATCAGCCCCAGCATTTT | 0 |
| AF043843 | TRBV12-4*02 | TGTGCCAGCAGTT.. | 2 | J1-5*01 | ......TCAGCCCCAGCATTTT | 6 |
| AF043844 | TRBV12-4*02 | TGTGCCAGCAGTT.. | 2 | J1-5*01 | ........AGCCCCAGCATTTT | 8 |
| AF043846 | TRBV12-4*02 | TGTGCCAGCAG.... | 4 | J1-5*01 | .......CAGCCCCAGCATTTT | 7 |
| AF079011 | TRBV28*01 | TGTGCCAGCAGTTT... | 3 | J1-5*01 | ......TCAGCCCCAGCATTTT | 6 |
| AF079054 | TRBV28*01 | TGTGCCAGCAGTTT... | 3 | J1-5*01 | .....ATCAGCCCCAGCATTTT | 5 |
| AJ549949 | TRBV3-1*01 | TGTGCCAGCAGCCAAGA | 0 | J1-5*01 | TAGCAATCAGCCCCAGCATTTT | 0 |
| S63251 | TRBV19*03 | TGTGCCAGTAGC | 0 | J1-5*01 | .......CAGCCCCAGCATTTT | 7 |
| M15564 | TRBV7-9*02 | TGTGCCAGCAGCTTA G | 0 | J1-5*01 | ....AATCAGCCCCAGCATTTT | 4 |
| AY082734 | TRBV2*01 | TGTGCCAGCAGTGAAGC | 0 | J1-6*01 | .......AATTCACCCCTCCACTTT | 7 |
| M64353 | TRBV6-6*01 | TGTGCCAGCAG...... | 6 | J1-6*02 | ..........TCACCCCTCCACTTT | 10 |
| AJ007775 | TRBV10-3*01 | TGTGCCATCAGTG.... | 4 | J1-6*01 | .......AATTCACCCCTCCACTTT | 7 |
| AF043785 | TRBV27*01 | TGTGCCAGCAGTTTAT. | 1 | J1-6*02 | .........TTCACCCCTCCACTTT | 9 |
| AF043786 | TRBV27*01 | TGTGCCAGCAGTTTAT. | 1 | J1-6*02 | .........TTCACCCCTCCACTTT | 9 |
| AY082730 | TRBV2*01 | TGTGCCAGC........ | 8 | J2-1*01 | ...CTACAATGAGCAGTTCTTC | 3 |
| AY006248 | TRBV4*01 | TCTGCGCCAGCAGCCAAGA | 0 | J2-1*01 | CTCCTACAATGAGCAGTTCTTC | 0 |
| AY006101 | TRBV6-6*01 | TGTGCCAGCAGT..... | 5 | J2-1*01 | CTCCTACAATGAGCAGTTCTTC | 0 |
| AY082714 | TRBV11*01 | TGTGCCAGCAGCTTAG. | 1 | J2-1*01 | ....TACAATGAGCAGTTCTTC | 4 |
| AY082715 | TRBV11*01 | TGTGCCAGCAGCT.... | 4 | J2-1*01 | ......CAATGAGCAGTTCTTC | 6 |
| AF043756 | TRBV28*01 | TGTGCCAGCA....... | 7 | J2-1*01 | CTCCTACAATGAGCAGTTCTTC | 0 |
| AF043757 | TRBV28*01 | TGTGCCAGCAGTTTA.. | 2 | J2-1*01 | ......CAATGAGCAGTTCTTC | 6 |
| AF043758 | TRBV28*01 | TGTGCCAGCA....... | 7 | J2-1*01 | .....ACAATGAGCAGTTCTTC | 5 |
| AF043759 | TRBV28*01 | TGTGCCAGCA....... | 7 | J2-1*01 | .........TGAGCAGTTCTTC | 9 |
| AF043787 | TRBV27*01 | TGTGCCAGCAGTTT... | 3 | J2-1*01 | ......CAATGAGCAGTTCTTC | 6 |
| AF043788 | TRBV27*01 | TGTGCCAGCAG...... | 6 | J2-1*01 | ......CAATGAGCAGTTCTTC | 6 |
| AF043789 | TRBV27*01 | TGTGCCAGCAG...... | 6 | J2-1*01 | ......CAATGAGCAGTTCTTC | 6 |
| AF043793 | TRBV19*03 | TGTGCCAGTAG. | 1 | J2-1*01 | ..........GAGCAGTTCTTC | 10 |
| AF043794 | TRBV19*03 | TGTGCCAGTAGC | 0 | J2-1*01 | ..CCTACAATGAGCAGTTCTTC | 2 |
| AF043795 | TRBV19*03 | TGTGCCAGTAG. | 1 | J2-1*01 | .........TGAGCAGTTCTTC | 9 |
| AF043809 | TRBV12-4*02 | TGTGCCAGCAGTTTA | 0 | J2-1*01 | ........ATGAGCAGTTCTTC | 8 |
| AF043810 | TRBV12-4*02 | TGTGCCAGCAGTTTA | 0 | J2-1*01 | ........ATGAGCAGTTCTTC | 8 |
| AF043819 | TRBV27*01 | TGTGCCAGCAGTTTAT. | 1 | J2-1*01 | ....TACAATGAGCAGTTCTTC | 4 |
| AF043820 | TRBV27*01 | TGTGCCAGTAG. | 1 | J2-1*01 | ....TACAATGAGCAGTTCTTC | 10 |
| AF043821 | TRBV27*01 | TGTGCCAGCAGT..... | 5 | J2-1*01 | ...CTACAATGAGCAGTTCTTC | 3 |
| AF043822 | TRBV27*01 | TGTGCCAGCAG...... | 6 | J2-1*01 | ....TACAATGAGCAGTTCTTC | 4 |
| AF043823 | TRBV27*01 | TGTGCCAGCAG...... | 6 | J2-1*01 | ....TACAATGAGCAGTTCTTC | 4 |
| AF043828 | TRBV19*03 | TGTGCCAGTAGC | 0 | J2-1*01 | .....ACAATGAGCAGTTCTTC | 5 |
| M13836 | TRBV9*01 | TGTGCCAGCAGCGTAG | 0 | J2-1*01 | ......CAATGAGCAGTTCTTC | 6 |
| X58327 | TRBV7-2*04 | TGTGCCAGCAGC... | 3 | J2-1*01 | .........TGAGCAGTTCTTC | 9 |
| Z77851 | TRBV9*03 | TGTGCCAGCAGC | 0 | J2-1*01 | ...CTACAATGAGCAGTTCTTC | 3 |
| AF043867 | TRBV19*03 | TGTGCCAGTAG. | 1 | J2-1*01 | ..........GAGCAGTTCTTC | 10 |
| AY082704 | TRBV11*01 | TGTGCCAGCAGCTT... | 3 | J2-2*01 | .....ACCGGGGAGCTGTTTTTT | 5 |
| AF043768 | TRBV12-4*02 | TGTGCCAGCAGTTT. | 1 | J2-2*01 | ....CACCGGGGAGCTGTTTTTT | 4 |
| AF043769 | TRBV12-4*02 | TGTGCCAGCAGT... | 3 | J2-2*01 | ....CACCGGGGAGCTGTTTTTT | 4 |
| AF043824 | TRBV27*01 | TGTGCCAGCAG...... | 6 | J2-2*01 | ........GGGGAGCTGTTTTTT | 8 |
| AF043825 | TRBV27*01 | TGTGCCAGCAG...... | 6 | J2-2*01 | ........GGGGAGCTGTTTTTT | 8 |
| AF043829 | TRBV19*03 | TGTGCCAGTAGC | 0 | J2-2*01 | .GAACACCGGGGAGCTGTTTTTT | 1 |
| AF043832 | TRBV28*01 | TGTGCCAGCAGTTTAT. | 1 | J2-2*01 | ..AACACCGGGGAGCTGTTTTTT | 2 |
| AF043833 | TRBV28*01 | TGTGCCAGCAGTTTAT. | 1 | J2-2*01 | ..AACACCGGGGAGCTGTTTTTT | 2 |
| AF043834 | TRBV28*01 | TGTGCCAGCAGTTTA.. | 2 | J2-2*01 | ....CACCGGGGAGCTGTTTTTT | 4 |
| AF043835 | TRBV28*01 | TGTGCCAGCAGTTTA.. | 2 | J2-2*01 | ...ACACCGGGGAGCTGTTTTTT | 3 |
| AF043847 | TRBV12-4*02 | TGTGCCAGCAGTTTA | 0 | J2-2*01 | ......CCGGGGAGCTGTTTTTT | 6 |
| AF043848 | TRBV12-4*02 | TGTGCCAGCAG.... | 4 | J2-2*01 | .....ACCGGGGAGCTGTTTTTT | 5 |
| AF043849 | TRBV12-4*02 | TGTGCCAGCAG.... | 4 | J2-2*01 | .....ACCGGGGAGCTGTTTTTT | 5 |
| AF043850 | TRBV12-4*02 | TGTGCCAGCAG.... | 4 | J2-2*01 | .GAACACCGGGGAGCTGTTTTTT | 1 |
| AF043868 | TRBV19*03 | TGTGCCAGTAG. | 1 | J2-2*01 | ...ACACCGGGGAGCTGTTTTTT | 3 |
| AF079057 | TRBV28*01 | TGTGCCA.......... | 10 | J2-2*01 | ....CACCGGGGAGCTGTTTTTT | 4 |
| AJ549953 | TRBV9*01 | TGTGCCAGCAGC.... | 4 | J2-2*01 | ...ACACCGGGGAGCTGTTTTTT | 3 |
| L34726 | TRBV4-3*01 | TGCGCCAGCAGCC.... | 4 | J2-2*01 | ..AACACCGGGGAGCTGTTTTTT | 2 |
| M13850 | TRBV5-6*01 | TGTGCCAGCAGCTTG. | 1 | J2-2*01 | .....ACCGGGGAGCTGTTTTTT | 5 |
| Z77838 | TRBV7-8*03 | TGTGCCAGCAGC | 0 | J2-2*01 | ......CCGGGGAGCTGTTTTTT | 6 |
| L34725 | TRBV27*01 | TGTGCCAGC........ | 8 | J2-2*01 | ......CCGGGGAGCTGTTTTTT | 6 |
| S67401 | TRBV4*01 | TCTGTGCCTGCAGCC.... | 4 | J2-3*01 | ......GATACGCAGTATTTT | 6 |
| M64354 | TRBV6-6*01 | TGTGCCAGC........ | 8 | J2-3*01 | AGCACAGATACGCAGTATTTT | 0 |
| AF043183 | TRBV28*01 | TGTGCCAGCAGTTT... | 3 | J2-3*01 | ..CACAGATACGCAGTATTTT | 2 |
| AF043790 | TRBV27*01 | TGTGCCAGCAGT..... | 5 | J2-3*01 | .GCACAGATACGCAGTATTTT | 1 |
| AF043801 | TRBV28*01 | TGTGCCAGCAG...... | 6 | J2-3*01 | ....CAGATACGCAGTATTTT | 4 |
| AF043811 | TRBV12-4*02 | TGTGCCAGCAGT... | 3 | J2-3*01 | ......GATACGCAGTATTTT | 6 |
| AF043812 | TRBV12-4*02 | TGTGCCAGCAGTTTA | 0 | J2-3*01 | ......GATACGCAGTATTTT | 6 |
| AF043826 | TRBV27*01 | TGTGCCAGCAGT..... | 5 | J2-3*01 | ......GATACGCAGTATTTT | 6 |
| AF043827 | TRBV27*01 | TGTGCCAGCAGTTTA.. | 2 | J2-3*01 | ......GATACGCAGTATTTT | 6 |
| AF043851 | TRBV12-4*02 | TGTGCCAGCAGTT.. | 2 | J2-3*01 | .GCACAGATACGCAGTATTTT | 1 |
| AF043859 | TRBV27*01 | TGTGCCAGCAG...... | 6 | J2-3*01 | ..CACAGATACGCAGTATTTT | 2 |
| AF043860 | TRBV27*01 | TGTGCCAGCAGTT.... | 4 | J2-3*01 | ...ACAGATACGCAGTATTTT | 3 |
| AF043861 | TRBV27*01 | TGTGCCAGCAGTT.... | 4 | J2-3*01 | ..CACAGATACGCAGTATTTT | 2 |
| AF043869 | TRBV19*03 | TGTGCCAGTAG. | 1 | J2-3*01 | ..CACAGATACGCAGTATTTT | 2 |
| AF043870 | TRBV19*03 | TGTGCCAGTAG. | 1 | J2-3*01 | ......GATACGCAGTATTTT | 6 |
| AF071472 | TRBV13*01 | TGTGCCAGCAGCTTAG. | 1 | J2-3*01 | ....CAGATACGCAGTATTTT | 4 |
| AF274827 | TRBV13*02 | TGTGCCAGCAGC | 0 | J2-3*01 | AGCACAGATACGCAGTATTTT | 0 |
| AJ417151 | TRBV30*05 | TGTGCC...... | 6 | J2-3*01 | AGCACAGATACGCAGTATTTT | 0 |
| AY239355 | TRBV7-3*01 | TGTGCCAGCAGC..... | 5 | J2-3*01 | AGCACAGATACGCAGTATTTT | 0 |
| M73464 | TRBV15*02 | TGTG.......... | 10 | J2-3*01 | ......GATACGCAGTATTTT | 6 |
| X86109 | TRBV19*03 | TGTGCCAGTAG. | 1 | J2-3*01 | ..CACAGATACGCAGTATTTT | 2 |
| X86115 | TRBV9*03 | TGTGCCAGCAGC | 0 | J2-3*01 | ..CACAGATACGCAGTATTTT | 2 |
| X86116 | TRBV7-7*02 | TGTGCCAGCAGC | 0 | J2-3*01 | AGCACAGATACGCAGTATTTT | 0 |
| Z77836 | TRBV30*05 | TGTGCCTGG... | 3 | J2-3*01 | ......GATACGCAGTATTTT | 6 |
| S60794 | TRBV6-6*01 | TGTGCCAGCAGTTAC.. | 2 | J2-3*01 | ..CACAGATACGCAGTATTTT | 2 |
| AY082733 | TRBV2*01 | TGTGCCAGCA....... | 7 | J2-4*01 | AGCCAAAAACATTCAGTACTTC | 0 |
| AY082735 | TRBV11*01 | TGTGCCAGCAGCTT... | 3 | J2-4*01 | ....AAAAACATTCAGTACTTC | 4 |
| AF043852 | TRBV12-4*02 | TGTGCCAGCAGT... | 3 | J2-4*01 | ......AAACATTCAGTACTTC | 6 |
| AY239354 | TRBV27*01 | TGTGCCAGCAG...... | 6 | J2-4*01 | .....AAAACATTCAGTACTTC | 5 |
| AF043813 | TRBV12-4*02 | TGTGCCAGCAGT... | 3 | J2-5*01 | .......GACCCAGTACTTC | 7 |
| AF043814 | TRBV12-4*02 | TGTGCCAGCAGT... | 3 | J2-5*01 | .......GACCCAGTACTTC | 7 |
| AF043836 | TRBV28*01 | TGTGCCAGCAGT..... | 5 | J2-5*01 | .......GACCCAGTACTTC | 7 |
| AF043853 | TRBV12-4*02 | TGTGCCAGCAGT... | 3 | J2-5*01 | .CCAAGAGACCCAGTACTTC | 1 |
| AF043854 | TRBV12-4*02 | TGTGCCAGCAGTT.. | 2 | J2-5*01 | .....GAGACCCAGTACTTC | 5 |
| AF043871 | TRBV19*03 | TGTGCCAGTAGC | 0 | J2-5*01 | ...AAGAGACCCAGTACTTC | 3 |
| AF071467 | TRBV9*03 | TGTGCCAGCAGC | 0 | J2-5*01 | .....GAGACCCAGTACTTC | 5 |
| AF079009 | TRBV28*01 | TGTGCCAGCAGTTTA.. | 2 | J2-5*01 | ...AAGAGACCCAGTACTTC | 3 |
| AF079010 | TRBV28*01 | TGTGCCAGCAG...... | 6 | J2-5*01 | .....GAGACCCAGTACTTC | 5 |
| AF079013 | TRBV28*01 | TGTGCCAGCAGT..... | 5 | J2-5*01 | ..CAAGAGACCCAGTACTTC | 2 |
| AF043815 | TRBV12-4*02 | TGTGCCAGCAG.... | 4 | J2-6*01 | .....GGGCCAACGTCCTGACTTTC | 5 |
| AF043855 | TRBV12-4*02 | TGTGCCAGCAGTTT. | 1 | J2-6*01 | CTCTGGGGCCAACGTCCTGACTTTC | 0 |
| AF043856 | TRBV12-4*02 | TGTGCCAGCAGTTTA | 0 | J2-6*01 | CTCTGGGGCCAACGTCCTGACTTTC | 0 |
| AF043857 | TRBV12-4*02 | TGTGCCAGCAG.... | 4 | J2-6*01 | .TCTGGGGCCAACGTCCTGACTTTC | 1 |
| AY082731 | TRBV2*01 | TGTGCCAGCAGTGAAG. | 1 | J2-7*01 | .....ACGAGCAGTACTTC | 5 |
| S60793 | TRBV3*01 | TGTGCCAGCAGCAAG. | 1 | J2-7*01 | ........AGCAGTACTTC | 8 |
| AY006107 | TRBV6-6*01 | TGTGCCAGCAGT..... | 5 | J2-7*01 | ..........CAGTACTTC | 10 |
| AB164057 | TRBV6-1*01 | TGTGCCAGCAG...... | 6 | J2-7*01 | ......CGAGCAGTACTTC | 6 |
| AF043181 | TRBV27*01 | TGTGCCAG......... | 9 | J2-7*01 | ...CTACGAGCAGTACTTC | 3 |
| AF043761 | TRBV28*01 | TGTGCCAGCAGT..... | 5 | J2-7*01 | ......CGAGCAGTACTTC | 6 |
| AF043762 | TRBV28*01 | TGTGCCAGCAG...... | 6 | J2-7*01 | .......GAGCAGTACTTC | 7 |
| AF043763 | TRBV28*01 | TGTGCCAGCAG...... | 6 | J2-7*01 | CTCCTACGAGCAGTACTTC | 0 |
| AF043796 | TRBV19*03 | TGTGCCAGTAGC | 0 | J2-7*01 | ...CTACGAGCAGTACTTC | 3 |
| AF043802 | TRBV28*01 | TGTGCCAGCAG...... | 6 | J2-7*01 | ..CCTACGAGCAGTACTTC | 2 |
| AF043816 | TRBV12-4*02 | TGTGCCAGCAGTTTA | 0 | J2-7*01 | ..CCTACGAGCAGTACTTC | 2 |
| AF043837 | TRBV28*01 | TGTGCCAGCAG...... | 6 | J2-7*01 | ...CTACGAGCAGTACTTC | 3 |
| AF043838 | TRBV28*01 | TGTGCCAGCAG...... | 6 | J2-7*01 | CTCCTACGAGCAGTACTTC | 0 |
| AF043839 | TRBV28*01 | TGTGCCAGCAGTT.... | 4 | J2-7*01 | ..CCTACGAGCAGTACTTC | 2 |
| AF043858 | TRBV12-4*02 | TGTGCCAGCAGT... | 3 | J2-7*01 | .......GAGCAGTACTTC | 7 |
| AF043862 | TRBV27*01 | TGTGCCAGCAG...... | 6 | J2-7*01 | .....ACGAGCAGTACTTC | 5 |
| AF043872 | TRBV19*03 | TGTGCCAGTAG. | 1 | J2-7*01 | .TCCTACGAGCAGTACTTC | 1 |
| AF043873 | TRBV19*03 | TGTGCCAGTAG. | 1 | J2-7*01 | ......CGAGCAGTACTTC | 6 |
| AF043889 | TRBV27*01 | TGTGCCAGCAGT..... | 5 | J2-7*01 | ...CTACGAGCAGTACTTC | 3 |
| M73465 | TRBV5-4*01 | TGTGCCAGCAGCTT.. | 2 | J2-7*01 | ..CCTACGAGCAGTACTTC | 2 |
| X68527 | TRBV5-4*01 | TGTGCCAGCAGCTT.. | 2 | J2-7*01 | ...CTACGAGCAGTACTTC | 3 |
| L34733 | TRBV19*01 | TGTGCCAGTAGT..... | 5 | J2-7*01 | ...CTACGAGCAGTACTTC | 3 |

1. Rowen, L., Koop, B.F. & Hood, L. The complete 685-kilobase DNA sequence of the human β T-cell receptor locus. *Science* **272**, 1755-1762 (1996).
