# Supplementary material for: Monitoring the T-Cell Receptor Repertoire at Single-Clone Resolution
Source: PLoS One. 2006 Dec 20;1(1):e55. doi: 10.1371/journal.pone.0000055 (PMC1762342; doi:10.1371/journal.pone.0000055)
Supplement: Table S2 — Maximum resolution for spectratyping and T-array protocol. (0.06 MB DOC) [file pone.0000055.s004.doc]

**TABLE S2. Maximum resolution for spectratyping and T-array protocol.** Protocols for repertoire analysis separate the repertoire in defined sub fractions. The number of separation units is given for each protocol.

**Table 2A. Vβ spectratyping**

| **Step in spectratyping protocol** | **Separation unit** | **Number of separation units** |
| --- | --- | --- |
| PCR amplification | Vβ - PCR product | 23* |
| Electrophoresis | PCR product of specific length^1^ | 8 to 10 |
|  | Total | 184 to 230 |

**Table 2B. Vβ/Jβ spectratyping**

| **Step in spectratyping protocol** | **Separation unit** | **Number of separation units** |
| --- | --- | --- |
| PCR amplification | Vβ/Jβ- PCR product | 276 |
| Electrophoresis | PCR product of specific length | 8 to10 |
|  | Total | 2208 to 2760 |

**Table 2C. T-array with Jβ annealer oligonucleotides**

| **Step in T-array protocol** | **Separation unit** | **Number of separation units** |
| --- | --- | --- |
| PCR amplification | Vβ-PCR product | 23* |
| Hybridization | Jβ-annealer | 144** |
| Ligation | Hexamer | 4,096*** |
|  | Total | 1.4∙10^7 ****^ |

|  |  |  |  |
| --- | --- | --- | --- |
|  |  |  |  |
|  |  |  |  |
|  |  |  |  |

***** 23 primers cover all functional Vβ families^2,3^

** 12 primers cover all functional Jβ families^2^. For 99% of the analyzed CDR3βs, a maximum number of 11 nucleotides is deleted from the germ line Jβ gene. For the T-array protocol this means that on average per Jβ family 12 different positions for labeled annealer oligonucleotides exist: 12 x 12 = 144.

*** A universal hexamer array contains 4^6^ = 4096 unique hexamer sequences.

**** If the TCRβ repertoire within one individual is estimated at 10^6^ unique sequences (*ref. 4*), on average 10^6^ / 1.4 ∙10^7^ = 0.07 CDRβ sequences will ligate to a single sequence on the universal microarray. This would create single-clone resolution.

1. Pannetier, C. *et al.* The sizes of the CDR3 hypervariable regions of the Murine T-cell receptor ß chains vary as a function of the recombined germ-Line segments.*,* *Proc. Natl. Acad. Sci. U.S.A.* **90**, 4319-4323 (1993).

2. Ruiz, M. *et al*. IMGT, the international ImMunoGeneTics database. *Nucleic Acids Res*. **28**, 219-221 (2000).

3. Doumaid, K. *et al*. Modulation of the T cell receptor beta chain repertoire after heart transplantation. *Transpl. Imm*. **8**, 83-94 (2000).

4. Arstila, T.P. *et al*. A direct estimate of the human alphabeta T cell receptor diversity. *Science* **286**, 958-961 (1999).
