# Supplementary material for: Monitoring the T-Cell Receptor Repertoire at Single-Clone Resolution
Source: PLoS One. 2006 Dec 20;1(1):e55. doi: 10.1371/journal.pone.0000055 (PMC1762342; doi:10.1371/journal.pone.0000055)
Supplement: Table S3 — Signal intensity T arrays. (0.12 MB DOC) [file pone.0000055.s005.doc]

**TABLE S3. Signal intensity T arrays.** The hexamer sequences of the strongest T-array signals are shown for T-cells stimulated with CMV-pp65 antigen. For further details see also the legend of Figure 5. Day 0 sample = before stimulation.

| **Exp.** | **Day 0** | | **Day 3** | | **Day 6** | |
| --- | --- | --- | --- | --- | --- | --- |
| **Rank** | **Sequence** | **Signal intensity** | **Sequence** | **Signal intensity** | **Sequence** | **Signal intensity** |
| 1 | CCTTTT | 2016 | CCTTTT | 10626 | CCTTTT | 18660 |
| 2 | CCGTTT | 1358 | CCCTTT | 8519 | CCGTTT | 4885 |
| 3 | TGGCTA | 1326 | CCTCTT | 3669 | CCTTCT | 4075 |
| 4 | GGACCG | 1239 | CCTTCT | 2601 | CCCTTT | 2647 |
| 5 | GTGCCG | 1009 | CCGTTT | 2281 | CCTTAT | 2043 |
| 6 | GTGCTA | 953 | CCTTAT | 2007 | CCTCTT | 1251 |
| 7 | CCTTAT | 929 | TCCTTT | 1898 | TCCTTT | 1243 |
| 8 | GGTCCG | 862 | TCTTTT | 1879 | CCATTT | 1140 |
| 9 | CCTTCT | 744 | ACATTT | 1863 | ACATTT | 1103 |
| 10 | GAGCCG | 711 | CCTGTT | 1733 | CCAGTT | 1058 |
| 11 | AGGGTA | 632 | CCGATT | 1687 | CATTTT | 763 |
| 12 | GGGCTA | 594 | ACCTTT | 1511 | CGTTTT | 725 |
| 13 | CAGGTA | 589 | CGTTTT | 1475 | CCTGTT | 629 |
| 14 | CGGCTA | 583 | CCATTT | 1463 | ACCTTT | 547 |
| 15 | AAGCTA | 580 | CCTATT | 1261 | GGTCCG | 528 |
| 16 | GGACTA | 543 | CATTTT | 1249 | CACTTT | 466 |
| 17 | CAGCTA | 537 | CTTTTT | 1156 | CTTTTT | 461 |
| 18 | GAGCTA | 531 | CCAGTT | 1138 | CCTTGT | 438 |
| 19 | TCCTTT | 483 | CCCATT | 1134 | CCCATT | 401 |
| 20 | CCCTTT | 467 | GCTTTT | 1127 | CCTTTC | 401 |
| 21 | GGAGTA | 467 | CCTTGT | 1116 | CCAATT | 322 |
| 22 | GGGCCG | 461 | CCTTTC | 1051 | CCGATT | 306 |
| 23 | GGATGA | 455 | TATTTT | 982 | GCTTTT | 276 |
| 24 | GGACAA | 454 | CACTTT | 978 | ATGTTT | 247 |
| 25 | TCTTTT | 453 | CATAGT | 949 | CATCTT | 228 |
| 26 | GTGGTA | 444 | ACTTCT | 940 | TCTTTT | 213 |
| 27 | GGACCA | 436 | GCCTCC | 932 | CTCTGT | 212 |
| 28 | GGGTCG | 427 | CTTAGG | 929 | CCCGCA | 209 |
| 29 | GCTTTT | 424 | ACAGCA | 922 | ACTTCT | 203 |
| 30 | GGTCTA | 423 | ACGTTT | 888 | ACTCTT | 190 |
| 31 | CCATTT | 419 | AAAGTA | 887 | CGATTT | 189 |
| 32 | ATGCTA | 418 | ACATGC | 883 | TCGTTT | 176 |
| 33 | GACAGA | 416 | AATCGG | 878 | CCCCTT | 170 |
| 34 | GCAGGA | 416 | TCATTT | 865 | CGCCGT | 163 |
| 35 | AGGTTA | 405 | CCCAGT | 851 | CCTATT | 162 |
| 36 | CAGGAA | 403 | CATGCC | 847 | CGTGCT | 158 |
| 37 | TTGCTA | 400 | TCCTCT | 839 | GGACCG | 158 |
| 38 | CATTTT | 392 | TCGTTT | 833 | ACTTAT | 156 |
| 39 | ACAGGA | 382 | AGTTTT | 831 | CATTAT | 151 |
| 40 | GGTCGG | 377 | TGGTGA | 826 | GAGCCG | 147 |
| 41 | TAGGGA | 373 | AAGAGT | 826 | ACTATT | 147 |
| 42 | CCTCTT | 363 | GCTCCA | 820 | GGGCCG | 138 |
| 43 | CAGGAG | 363 | AAAACA | 819 | CAGCTT | 137 |
| 44 | GAGATG | 355 | TCGGTA | 811 | CTATCT | 122 |
