# Supplementary material for: Monitoring the T-Cell Receptor Repertoire at Single-Clone Resolution
Source: PLoS One. 2006 Dec 20;1(1):e55. doi: 10.1371/journal.pone.0000055 (PMC1762342; doi:10.1371/journal.pone.0000055)
Supplement: Table S4 — Comparing T-array signals to results from cloning and sequencing in the ex vivo CMV-stimulation experiment. (0.29 MB DOC) [file pone.0000055.s006.doc]

**TABLE S4. Comparing T-array signals to results from cloning and sequencing**

**in the ex vivo CMV-stimulation experiment**

**(A-C)** The clonal frequencies estimated by cloning and sequencing Vβ13^+^/ Jβ1-2^+^ PCR products are compared to T-array signals at (A) day 0, (B) day 3 and (C) day 12 (See figure 4). The sequence ACTATGGCT.. shows the 5’-end of the annealer oligonucleotide that was used in the T-array experiment, and is identical to the germline Jβ1-2 sequence with three nucleotides deleted.

TCR sequences with more than 3 nucleotides deleted from this germ-line sequence were picked up by sequencing. However, these cannot be detected by the T-array using an annealer that has only 3 nucleotides deleted (See Figure 4), and are not shown in these tables. As consequence sequence information is only shown for 27 out of 52 clones at day 0, 40 out of 46 at day 3 and 19 out of 22 clones at day 12. **(D)** The T-array data at day 0 identify 6 clones, which are confirmed by cloning and sequencing. Other hexamer sequences that give high signals (Table S3, Column Day 0) are highly similar to the sequences shown, indicating these are a result of cross ligation.

.

**A. CDR3s Vβ13^+^/ Jβ1-2^+^ T-cell clones Day 0**

| **Sequence** | **Frequency** | **Joining sequence**  **N - Jβ** | **Signal**  **T-array** |
| --- | --- | --- | --- |
| CASSDLGTSFYYGYTF  CASSSRQGADYGYTF  CASSSLPGQGNYGYTF  CASSPDAGNYGYTF  CASSPERKIANYGYTF  CASSLTGTANYGYTF  CASSQEYRTDGYTF  CASSYSRGWDYGYTF  CASSPDRSPTDYGTF  CASSYGNYGYTF  CASRQ*ANYGYTF  CASSVTGDYGYTF  CASSYWVQGPVDYGYTF  CASSYQGQLNYGYTF | 7/52  7/52  1/52  1/52  1/52  2/52  1/52  1/52  1/52  1/52  1/52  1/52  1/52  1/52 | CCTTTTACTATGGCT..  GGACCGACTATGGCT..  AGGGTAACTATGGCT..  CAGGTAACTATGGCT..  AAGCTAACTATGGCT..  CAGCTAACTATGGCT..  GGACAGACTATGGCT..  GCTGGGACTATGGCT..  CTACGGACTATGGCT..  CTGTGGACTATGGCT..  AAGCGAACTATGGCT..  CAGGGGACTATGGCT..  ATGGAAACTATGGCT..  AGCTAAACTATGGCT.. | 2016  1239  632  589  580  537  254  94  73  41  25  7  0  0 |

**B. CDR3s Vβ13^+^/ Jβ1-2^+^** T-cell clones Day 3

| **Sequence** | **Frequency** | **Joining sequence**  **N - Jβ** | **Signal**  **T-array** |
| --- | --- | --- | --- |
| CASSDLGTSFYYGYTF  CASSLTGTANYGYTF | 39/46  1/46 | CCTTTTACTATGGCT..  CAGCTAACTATGGCT.. | 10612  643 |

**C. CDR3s Vβ13^+^/ Jβ1-2^+^T-cell clones Day 12**

| **Sequence** | **Frequency** | **Joining sequence**  **N - Jβ** |
| --- | --- | --- |
| CASSDLGTSFYYGYTF | 19/22 | CCTTTTACTATGGCT.. |

**D. T-array of the day 0 sample.**
