# Supplementary material for: Monitoring the T-Cell Receptor Repertoire at Single-Clone Resolution
Source: PLoS One. 2006 Dec 20;1(1):e55. doi: 10.1371/journal.pone.0000055 (PMC1762342; doi:10.1371/journal.pone.0000055)
Supplement: Table S5 — T-array analysis of CMV-specific cells in vivo. (0.07 MB DOC) [file pone.0000055.s007.doc]

**TABLE S5. T-array analysis of CMV-specific cells *in vivo*.** The hexamer sequences of the strongest T-array signals are shown for T-cells isolated from a renal transplant patient during the height of the CMV-PCR peak. For further details see also the legend of Figure 6.

|  | **Vβ6.1- Jβ2.7 T-array** | |
| --- | --- | --- |
| **Rank** | **Sequence** | **Signal** |
| 1 | CGGCTC | 58164 |
| 2 | GAGGAA | 35121 |
| 3 | CCAGTC | 13087 |
| 4 | AGGCTC | 11561 |
| 5 | GGGCTC | 10731 |
| 6 | CGGATC | 10430 |
| 7 | TGGCTC | 8638 |
| 8 | CGGGTC | 8508 |
| 9 | CGGCCC | 6792 |
| 10 | GACCTT | 6129 |
| 11 | CGGTTC | 5946 |
| 12 | CACGGG | 5886 |
| 13 | CGGCAC | 4687 |
| 14 | AAGGAA | 4575 |
| 15 | GTATTC | 4333 |
| 16 | GGCTCA | 4276 |
| 17 | GAGGCA | 4064 |
| 18 | CGGCGC | 3656 |
| 19 | ACAGTC | 3236 |
| 20 | GAGGGA | 3037 |
| 21 | CCGGTC | 2895 |
| 22 | GCGGAA | 2622 |
| 23 | CCCGTC | 2605 |
| 24 | GAGGAG | 2580 |
| 25 | CAGGAA | 2559 |
| 26 | CGGTCC | 2548 |
| 27 | CTGCTC | 2408 |
| 28 | GCAGTC | 2314 |
| 29 | TGGGTC | 2201 |
| 30 | CCAGCC | 2106 |
| 31 | GGCGAA | 2094 |
| 32 | GTGGAA | 2053 |
| 33 | CCGCTC | 2031 |
| 34 | GATGAA | 1969 |
| 35 | CGTAGC | 1941 |
| 36 | AGGAGA | 1907 |
| 37 | GGACAA | 1900 |
| 38 | AGGTTC | 1873 |
| 39 | GAGTAA | 1834 |
| 40 | ACGGAA | 1790 |
| 41 | GAGGTA | 1760 |
| 42 | TAGGAA | 1677 |
| 43 | TCGGTC | 1653 |
| 44 | GGACTA | 1649 |
| 45 | CTGGTC | 1615 |
